# The D84G mutation in STIM1 causes nuclear envelope dysfunction and myopathy in mice

**DOI:** 10.1172/JCI170317

**Published:** 2024-02-01

**Authors:** Victoria Bryson, Chaojian Wang, Zirui Zhou, Kavisha Singh, Noah Volin, Eda Yildirim, Paul Rosenberg

**Affiliations:** 1 Department of Medicine,; 2 Duke Cardiovascular Research Center,; 3 Department of Cell Biology,; 4 Duke Cancer Institute, Duke University Medical Center, and; 5 Duke Molecular Physiology Institute, School of Medicine, Durham, North Carolina, USA.

**Keywords:** Cell biology, Muscle biology, Calcium signaling, Genetic diseases

## Abstract

Stromal interaction molecule 1 (STIM1) is a Ca^2+^ sensor located in the sarcoplasmic reticulum (SR) of skeletal muscle, where it is best known for its role in store-operated Ca^2+^ entry (SOCE). Genetic syndromes resulting from STIM1 mutations are recognized as a cause of muscle weakness and atrophy. Here, we focused on a gain-of-function mutation that occurs in humans and mice (STIM1^+/D84G^ mice), in which muscles exhibited constitutive SOCE. Unexpectedly, this constitutive SOCE did not affect global Ca^2+^ transients, SR Ca^2+^ content, or excitation-contraction coupling (ECC) and was therefore unlikely to underlie the reduced muscle mass and weakness observed in these mice. Instead, we demonstrate that the presence of D84G STIM1 in the nuclear envelope of STIM1^+/D84G^ muscle disrupted nuclear-cytosolic coupling, causing severe derangement in nuclear architecture, DNA damage, and altered lamina A–associated gene expression. Functionally, we found that D84G STIM1 reduced the transfer of Ca^2+^ from the cytosol to the nucleus in myoblasts, resulting in a reduction of [Ca^2+^]_N_. Taken together, we propose a novel role for STIM1 in the nuclear envelope that links Ca^2+^ signaling to nuclear stability in skeletal muscle.

## Abstract 2

The D84G STIM1 mutation within the nuclear envelope disrupts nuclear-cytosolic coupling causing nuclear instability via reduced calcium transfer in myoblasts.

## Introduction

Stromal interaction molecule 1 (STIM1) is a transmembrane protein located in the sarcoplasmic reticulum (SR) of skeletal muscle, where it senses Ca^2+^ stores and activates Orai channels as part of store-operated Ca^2+^ entry (SOCE). SOCE in muscle fibers is required for Ca^2+^ homeostasis and to sustain Ca^2+^ transients during neuromuscular activity (1). Loss-of-function mutations in the human and mouse genes for STIM1 and calcium release–activated calcium modulator 1 (Orai1) cause reduced muscle growth and weakness, in addition to severe combined immune deficiency (2–4). Gain-of -function mutations in STIM1 and Orai1 are increasingly recognized as a genetic syndrome that includes hyposplenism, platelet bleeding diathesis, and tubular aggregate myopathy (TAM1) (5). Tubular aggregates form in specific regions of the SR membrane that contain STIM1, calsequestrin (CASQ), and SERCA1 but exclude the ryanodine receptor 1 (RYR1). While tubular aggregates are associated with STIM1 mutations, it remains to be determined if they are required for STIM1 myopathy. In fact, various mouse models of the STIM1 mutations fail to manifest tubular aggregates but do show weakness (6–10).

In skeletal muscle, the SR is composed of highly specialized regions that include the junctional cisternae (jSR), the longitudinal SR (lSR), and the perinuclear membrane. The lSR houses the RYR1, is located adjacent to the A-I bands of the sarcomere, and is the site of Ca^2+^ release during excitation contraction coupling (ECC). The lSR encompasses the fenestrated membrane near the Z-line that is enriched in sarcoplasmic/endoplasmic reticulum Ca^2+^-ATPase, (SERCA1) and functions in Ca^2+^ store refilling, protein synthesis, and protein secretion. STIM1 is organized into operational domains of the SR membrane that include the lSR and the jSR. STIM1 in the jSR interacts with Orai1 in the T-tubular membrane to activate SOCE. We recently showed that STIM1 is tethered in the lSR by the type III intermediate filament (IF) desmin to regulate SERCA1 and Ca^2+^ signaling in the muscle fibers (11). Desmin anchors STIM1 to the Z-disc of muscle fibers, regulates SOCE, and prevents Ca^2+^ overload by facilitating store refilling by SERCA1 pumps.

In contrast to our understanding of STIM1 in the SR membrane, far less is known about the role STIM1 has in the nuclear membrane (12). STIM1 can be detected in the outer nuclear membrane and as part of the nuclear reticulum. We hypothesized that STIM1 has an essential role in nuclear-cytosolic connectivity and interacts with proteins of the linker of nucleoskeleton and cytoskeleton (LINC) complex. The LINC complex is composed of several proteins that span the nuclear envelope and include lamin A/C in the nuclear lamina, Sad1 and UNC84 domain–containing proteins (SUN1/2) of the inner nuclear membrane (INM), and Kash/Nesprin proteins of the outer nuclear membrane (ONM) (13). Compromise of the nuclear-cytosolic connectivity can influence nuclear morphology, induce apoptosis, and influence gene expression, as occurs in Emery Dreifuss muscular dystrophy (EDMD) and other laminopathies (14).

In the present work, we used a mouse model bearing a mutation in the EF hand (helix-loop-helix structural domain) (D84G) of STIM1 to explore the role of STIM1 in the nuclear membrane in vivo (15, 16). We show that STIM1^+/D84G^ mice exhibited constitutively activated SOCE but had muscle fibers that were spared the consequence of Ca^2+^ overload due to remodeling of Ca^2+^ handling apparatus in these muscles. Intriguingly, we found that changes in the nuclear morphology that resembled changes observed in myopathies associated with laminopathies. These findings offer insight into the pathogenesis of STIM1 myopathies and demonstrate a previously unrecognized role for STIM1 in the nuclear envelope, gene expression, and DNA damage.

## Results

### Reduced muscle mass in STIM1^+/D84G^ mice.

**Figure 1:**
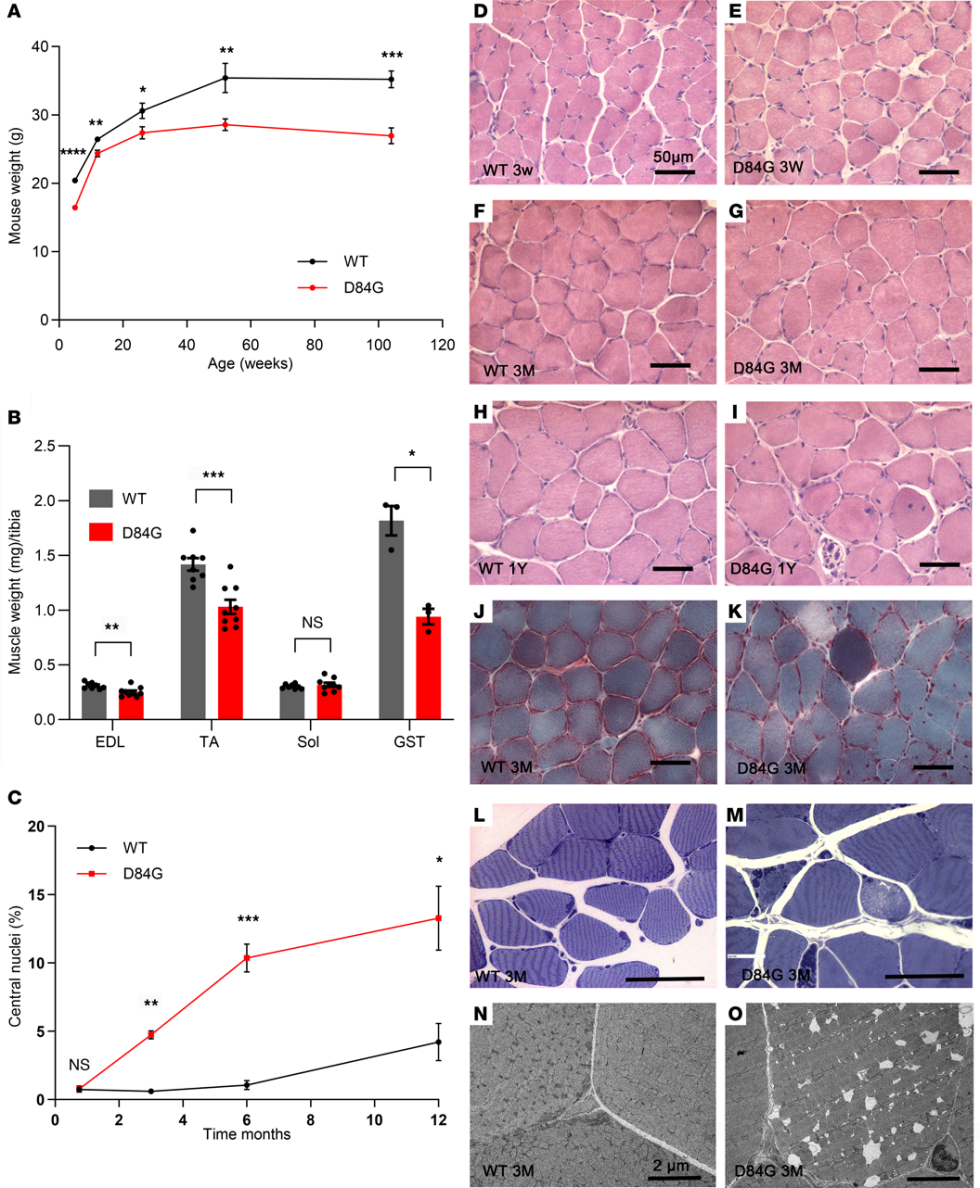
Characterization of STIM1^+/D84G^ muscle. (**A**) Mouse weight with time (*n =* 6–9 male mice per genotype). *P <* 0.0228, by 2-way ANOVA. (**B**) Muscle weight/tibia length of EDL, Sol, TA, and GST muscle from 6-month-old WT and STIM1^+/D84G^ mice (*n =* 3–9 per genotype) (**C**) Quantification of the central nuclei percentage in Sol muscle from WT and STIM1^+/D84G^ mice (*n =* 3) at 3 weeks to 1 year of age. *P <* 0.0005, by ANOVA. (**D**–**I**) H&E staining of 14 μm cryosections of Sol muscle from (**D**) 3-week-old (3W) WT, (**E**) 3-week-old D84G, (**F**) 3-month-old WT (3M) (**G**), 3-month-old D84G (**H**), 1-year-old (1Y) WT, and (**I**) 1-year-old D84G 1 mice (*n =* 3). (**J** and **K**) GÖmÖri trichrome staining of Sol muscle sections from 3-month-old (**J**) WT and (**K**) D84G mice (*n >*3). (**L** and **M**) Toluidine blue staining of thin, resin-embedded sections of (**L**) WT and (**M**) D84G TA muscle. (**N** and **O**) TEM micrographs of ultrathin TA muscle sections from (**N**) WT and (**O**) D84G mice (*n >*3). Values are the mean ± SEM. *P < 0.05, **P < 0.01, ***P < 0.001, and ****P < 0.0001, by 2-tailed Student’s *t* test (NS, *P >* 0.05).

STIM1^+/D84G^ mice are fertile and exhibit no excess mortality compared with their WT littermates. We did, however, observe a significant reduction in body weight in a cohort of STIM1^+/D84G^ mice (Figure 1A). In mice at 6 months of age, we observed a reduction in muscle mass (normalized to tibia length) for specific muscles including tibialis anterior (TA), extensor digitorum longus (EDL), and gastrocnemius (GST) muscles (Figure 1B). The mass of the soleus (Sol) muscles did not differ (Figure 1B), and tibia length did not differ for STIM1^+/D84G^ or WT mice (Supplemental Figure 1A; supplemental material available online with this article; https://doi.org/10.1172/JCI170317DS1). We found a significant reduction in the cross-sectional area of type IIB muscle fibers relative to type IIA fibers in the EDL muscles of STIM1^+/D84G^ mice (Supplemental Figure1B). These data suggest that the loss of muscle mass for STIM1^+/D84G^ mice selectively involved muscles enriched in fast glycolytic fibers, whereas muscles enriched in slow oxidative fibers maintained muscle mass. In fact, we noted a significant shift in fibers from the type IIB to type IIX based on immunofluorescence for the different myosin heavy chain isoforms as well as RNA expression (Supplemental Figure 1, C and D).

### Histopathology of the STIM1^+/D84G^ muscle.

Histopathology for STIM1^+/D84G^ skeletal muscles (Sol, EDL, and GST), evaluated by H&E-staining of sections, revealed a progressive increase in the number of central nuclei over 1 year in the Sol muscle sections (Figure 1, C–I). The EDL and GST muscle sections were analyzed at 6 months, and STIM1^+/D84G^ mice were found to have increased central nuclei as compared with their WT littermates (Supplemental Figure 1, E–I). GÖmÖri staining of muscle sections from STIM1^+/D84G^ mice showed that muscle fibers were variable in size and color, ranging from pale necrotic fibers to intensely stained fibers, consistent with excess contractile damage (Figure 1, J and K). However, GÖmÖri did not reveal changes to the SR that are typically seen for tubular aggregates. Staining of the SR for SERCA1 confirmed that there was no increase in tubular aggregate formation. In fact, there was a trend toward fewer tubular aggregates in the muscles of STIM1^+/D84G^ mice (Supplemental Figure 1J). Toluidine blue–stained sections from 3-month-old STIM1^+/D84G^ mice identified abnormally shaped, pale fibers, necrotic fibers, and expansion of the extracellular matrix — nonspecific findings often seen in skeletal myopathies (Figure 1, L and M). TA muscles from 3-month-old WT and STIM1^+/^^D84G^ mice were examined by transmission electron microscopy (TEM) (Figure 1, N and O. We noted structural changes in muscle sections from STIM1^+/D84G^ mice that included marked dilation of the terminal cisternae of the SR, which resembled the steps described for tubular aggregate development in aged muscles, where SR dilation precedes tubular aggregate formation (17).

### Muscle-specific gene expression profiling of D84G mice.

**Figure 2:**
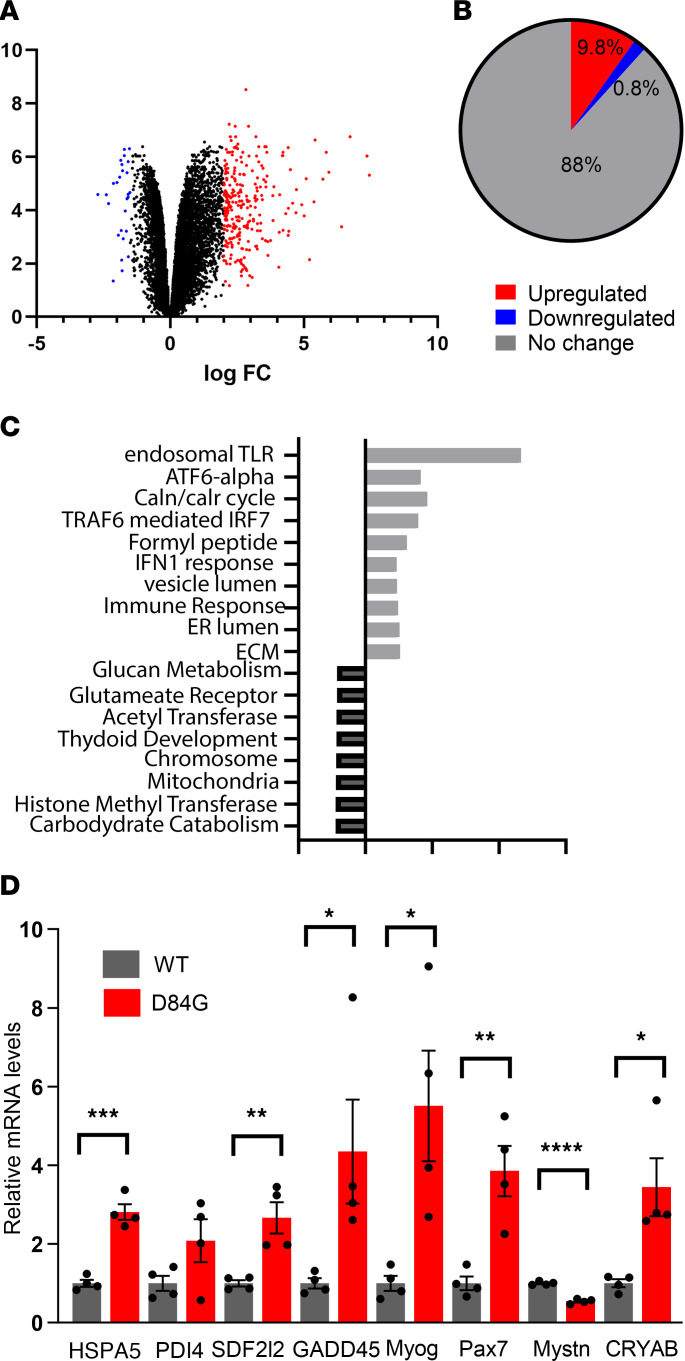
RNA-Seq for GST muscles from 6-month-old WT and STIM1^+/D84G^ mice. mRNA from 6-month-old WT (*n =* 4 female) and STIM1^+/D84G^ (*n =* 4 female) mice was prepared from GST muscle. (**A**) Volcano plot for DEGs from WT and STIM1^+/D84G^ mice. Red dots represent upregulated genes; blue dots represent downregulated genes. (**B**) Graphic representation of DEGs that were upregulated or downregulated. (**C**) GO pathway analysis for DEGs. The top pathways for upregulated DEGs (black) and downregulated DEGs (gray) are shown. (**D**) Quantification of ER stress gene expression in STIM1^+/D84G^ mouse muscle using RT-PCR. Values are the mean ± SD. Relative mRNA levels were normalized to *Gapdh*. *n ≥*4 independent experiments. *P < 0.05, **P < 0.01, ***P < 0.001, and ****P < 0.0001, by 2-tailed Student’s *t* test (NS, *P >* 0.05).

To understand the differences in gene expression between muscles from WT and STIM1^+/D84G^ mice, we performed RNA-Seq in GST muscles isolated from 6-month-old mice. The volcano plots show differentially expressed genes (DEGs) in STIM1^+/D84G^ mice that included 15,434 transcript reads. Of the total transcripts, 1,543 DEGs (~10%) were upregulated and only 116 DEGs (~1.8%) were downregulated, based on a statistical cutoff of the normalized enrichment score (NES >2.5) and a FDR of less than 0.05 (Figure 2, A and B, and Supplemental Table 1).

### Pathway analysis for RNA-Seq in skeletal muscle from STIM1^+/D84G^ mice.

Gene Ontology (GO) analysis identified significant changes in several biologic processes and pathways for the upregulated DEGs that associate with inflammation (TLR, IFN signaling, type I immune response), ER stress (ATF-6, calnexin, ER lumen), myogenesis, and extracellular matrix (Figure 2C). Involvement of the ER stress and unfolded protein pathways was validated by quantifying the mRNA by quantitative PCR (qPCR) for heat shock protein family A (HSP70), member 5 (HSPA5), protein disulfide isomerase family A member 4 (PDIA4), growth arrest and DNA damage–inducible α (GADD45), and stromal cell–derived factor 2–like 1 (SDF2L1). ER stress pathways sense accumulation of the misfolded protein and activate transcriptional pathways that upregulate chaperones in the ER to promote refolding (Figure 2D). We also noted upregulation of myogenic factors, including myogenin (MYOG), paired box 7 (PAX7), myostatin (MSTN), and aβ-crystallin (CRYAB).

On the basis of the DEGs downregulated in the muscles of STIM1^+/D84G^ mice, GO enrichment tools identified signaling pathways associated with carbohydrate catabolism, histone methyltransferase activity, mitochondrial oxidative metabolism, DNA repair, chromosome organization, and chromatin modification (Figure 2C). These pathways are frequently linked to chromatin organization, muscle differentiation, and mitochondrial biogenesis. Taken together, marked changes in the transcriptome of muscles from STIM1^+/D84G^ mice led to changes in pathways related to ER stress and upregulated immune responses, whereas pathways associated with chromatin regulation were downregulated.

### Spontaneous Ca^2+^ entry into the D84G muscle fibers.

**Figure 3:**
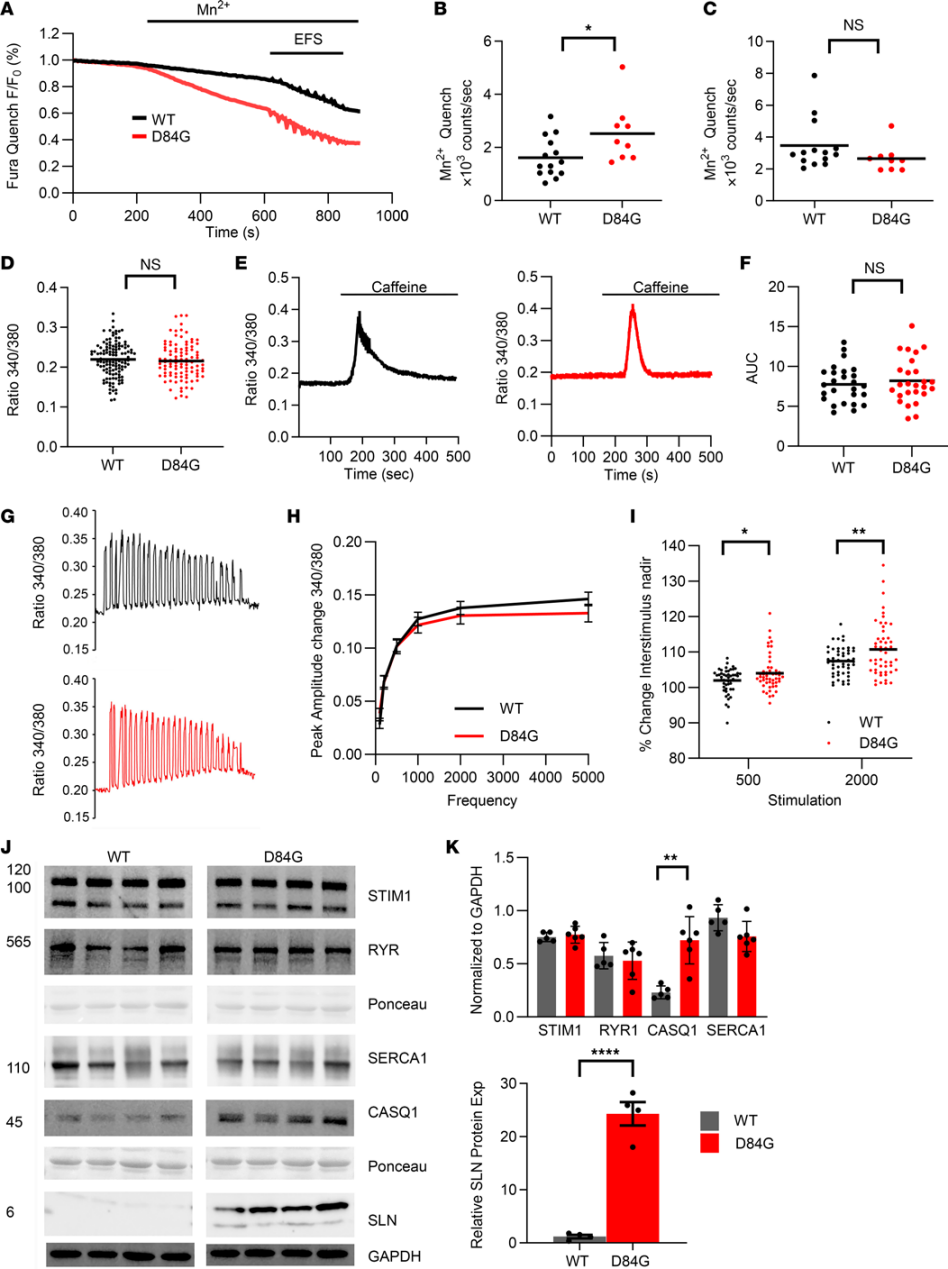
Ca^2+^ signaling in D84G-mutant mice. (**A**–**C**) Manganese (Mn^2+^) quench assays were performed on Fura-2–loaded FDB fibers to quantitate SOCE expression in WT and STIM1^+/D84G^ mice (*n =* 3 male mice; *n* = 30 fibers) (**A**) Spontaneous and EFS Mn^2+^ quench rate in WT and STIM1^+/D84G^ fibers. (**B**) Spontaneous Mn^2+^ quench rate was significantly increased in STIM1^+/D84G^ mice (*P* > 0.05). (**C**) The Mn^2+^ quench following electrical field stimulation (EFS) was not significantly different. (**D**) Basal Ca^2+^ levels measured by the Fura-2 method were not different between WT and STIM1^+/D84G^ fibers. (**E** and **F**) Ca^2+^ release evoked by caffeine from Fura-4F–loaded FDB fibers (**E**). The AUC (**F**) did not differ between WT and STIM1^+/D84G^ fibers (*n =* 3 mice; *n* = 30 fibers per genotype). (**G**) EFS Ca^2+^ transients from Fura-4F–loaded FDB muscle fibers were similar (*n =* 4 mice; *n* = 50 fibers per genotype). (**H** and **I**) Graphical representation of peak amplitude and frequency (**H**) shows no significant changes in peak Ca^2+^ release per EFS (**I**) but a significant elevation in interstimulus Ca^2+^ from STIM1^+/D84G^ fibers (*n* = 3 mice; *n =* 50 fibers per genotype). (**J** and **K**) Western blot analyses of Ca^2+^ handling proteins in 6-month-old WT and STIM1^+/D84G^ mice (*n =* 5 per genotype). Lysates from GST muscles were prepared from WT and STIM1^+/D84G^ mice. Antibodies for STIM1, RYR1, SERCA1, CASQ (top), and SLN (bottom) were used to quantify protein expression (Exp) versus GAPDH. Values are the mean ± SD. *n ≥*4 independent experiments. *P < 0.05, **P < 0.01, and ****P < 0.0001, by 2-tailed Student’s *t* test (NS, *P >* 0.05).

The N-terminus of STIM1 extends into the SR lumen, where EF hands sense the depletion of luminal Ca^2+^ stores (18). The D84G mutation in STIM1 disrupts the globular compact structure created by the EF hand and SAM domains, thereby reducing the Ca^2+^ bound to it. As shown by others, this mutant STIM1 associates with Orai1 channels in the absence of store depletion and confers spontaneous Ca^2+^ entry (19). To examine the effect of D84G STIM1 on Ca^2+^ entry into flexor digitorum brevis (FDB) muscle fibers, we performed manganese (Mn^2+^) quenching assays by exciting Fura-2AM (360 nm) at the isosbestic wavelength. Manganese (1.8 mM) application to the cellular bath solution is detected as a loss of the emission of Fura-2 signal and thereby represents cationic flux across the sarcolemma. This enables the separation of Ca^2+^ entry and Ca^2+^ release and is known to be linearly related to SOCE. Addition of Mn^2+^ (1.8 mM) to D84G fibers reduced the fluorescence signal in the absence of store depletion to a greater degree than that seen in WT fibers, consistent with increased spontaneous Ca^2+^ entry into D84G muscle (Figure 3, A and B). SOCE is activated during neuromuscular activity of WT fibers by both tonic and phasic patterns (20). Electrical field stimulation (EFS) of WT fibers led to a greater rate of Fura-2 quenching compared with the rate in the absence of EFS within the same fiber, which was consistent with SOCE phasic activation (Figure 3, A and B). Importantly, no differences in the rate of Fura-2 quenching were detected in the EFS-stimulated WT and STIM1^+/D84G^ fibers (Figure 3, A and C). These data suggest that D84G STIM1 can spontaneously activate Orai1 channels in the T-tubular membrane. Because the STIM1^+/D84G^ fibers also contain WT STIM1, STIM1 Ca^2+^ sensor function was intact, as SOCE was activated by EFS. Despite the spontaneous Ca^2+^ entry into D84G fibers, we observed no differences in basal Ca^2+^ levels (Figure 3D). In addition, Ca^2+^ release from internal stores induced by caffeine (a RYR1 activator) and SERCA inhibition by cyclopiazonic acid (CPA) was detected in STIM1^D84G^ and WT mouse fibers (Figure 3, E and F).

To understand how the D84G STIM1 mutation influences EFS-evoked Ca^2+^ transients, we measured transients from Fura-4–loaded FDB fibers (21). We detected no difference in peak amplitude of the Ca^2+^ transients over a range of stimulation frequencies (1–50 Hz) in WT or STIM1^+/D84G^ fibers (Figure 3, G and H). However, analysis of the interstimulus nadir of Ca^2+^ revealed an increase in Ca^2+^ for STIM1^+/D84G^ fibers (Figure 3, G and I). Specifically, the change in Ca^2+^ was apparent after 500 seconds and 2,000 seconds of EFS. These data may represent the spontaneous SOCE that persists in the stimulated fiber. From these data it appears that the presence of D84G STIM1 in the SR did not significantly alter the Ca^2+^ transients. We therefore scrutinized RNA-Seq data from STIM1^+/D84G^ muscle for changes in the expression of factors that regulate Ca^2+^ handling proteins. Sarcolipin (SLN), an endogenous muscle-specific inhibitor of the SERCA1 pump, was among the most dramatically upregulated mRNAs (~40-fold) in STIM1^+/D84G^ muscle, with a corresponding increase in SLN protein levels (Figure 3, J and K) (22). STIM1, SERCA1, and RYR1 expression levels were unchanged in GST muscles of STIM1^+/D84G^ mice (Figure 3, J and K). In contrast, the SR-Ca^2+^ buffering protein CASQ1 was downregulated in the RNA-Seq data, but the level of CASQ1 protein was significantly increased in STIM1^+/D84G^ muscle lysates. Taken together, these changes in Ca^2+^ handling proteins, such as SLN and CASQ1, likely represent an important adaptive mechanism available to muscle fibers that protects fibers against Ca^2+^ store overload that would otherwise be expected when SOCE is constitutive.

### Impaired exercise capacity for D84G mice.

**Figure 4:**
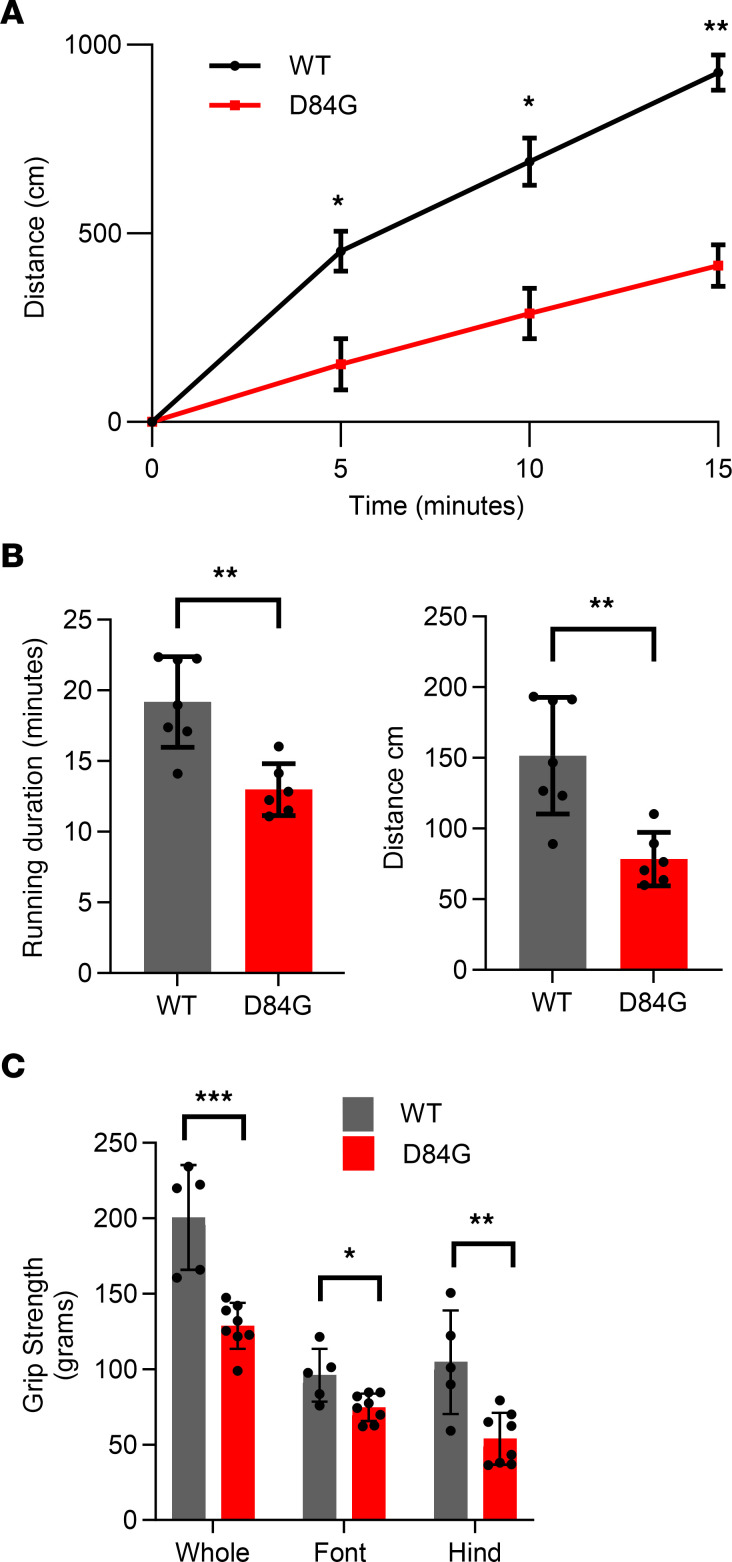
Muscle performance is reduced in the STIM1^+/D84G^ mice. (**A**) Spontaneous locomotion was quantified using an exploratory open-field platform. Recordings of movement every 5 minutes were quantified for WT and STIM1^+/D84G^ mice. Male mice were tested at 3 months (left) and 1 year (right) (*n =* 3 mice for each genotype and age). (**B**) Treadmill running duration (left) and distance (right) for 3-month-old WT (*n =* 6) and STIM1^+/D84G^ mice (*n =* 6). (**C**) Grip strength was measured for the front, rear, and whole body (*n =* 6). Values are the mean ± SD. *n ≥*6 independent experiments. *P < 0.05, **P < 0.01, and ***P < 0.001, by 2-tailed Student’s *t* test (NS, *P >* 0.05).

To assess general locomotor activity for STIM1-mutant mice, we assessed open-field exploratory behavior of WT and STIM1^+/D84G^ mice. The distance explored by STIM1^+/D84G^ mice was significantly shorter than for their WT counterparts (Figure 4A). WT mice progressively explored their environment over the entire 15-minute interval. However, there was little cumulative movement of the STIM1^+/D84G^ mice. These data identify a progressive decline in locomotor activity for STIM1^+/D84G^ mice over time compared with WT mice. We next assessed the maximal exercise capacity of 3-month-old STIM1^+/D84G^ mice in vivo using a standard treadmill running protocol (Figure 4B). The significant reduction in time to exhaustion and running distance for STIM1^+/D84G^ mice demonstrates that these mice had limited exercise capacity compared with WT mice (Figure 4A). Grip strength was also significantly reduced for 6-month-old STIM1^+/D84G^ mice compared with WT mice (Figure 4C). Despite relatively normal Ca^2+^ transients, the STIM1^+/D84G^ mice experienced a progressive decline in locomotor function and reduced exercise capacity.

### STIM1 in the nuclear membrane.

**Figure 5:**
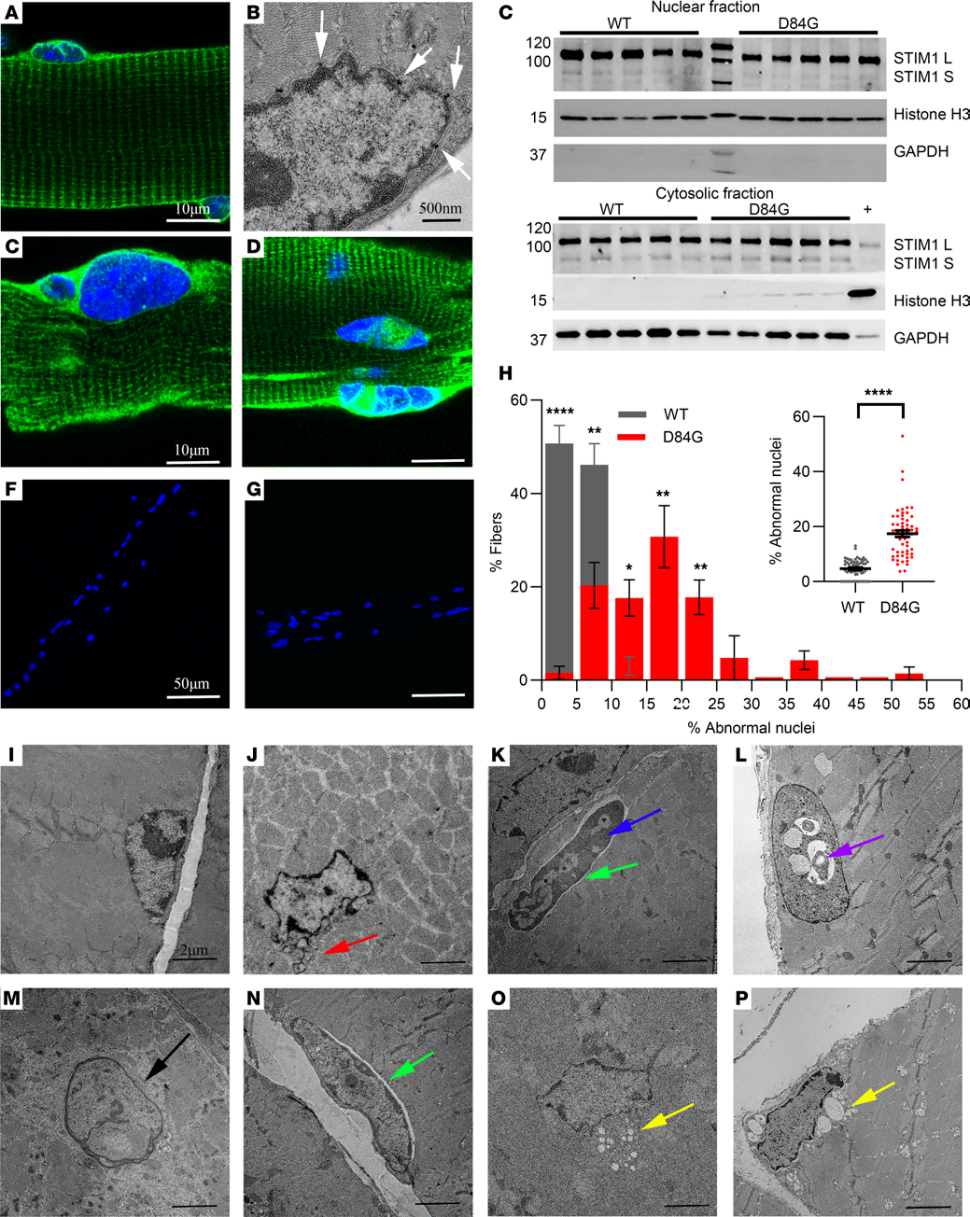
Nuclear abnormalities in muscles of STIM1^+/D84G^ mice. (**A**) Immunostaining for STIM1 (green) and nuclei (DAPI, blue) in WT mice (male and female) (*n >*6). Scale bar: 10 μm. (**B**) Electron micrograph showing expression of STIM LacZ (white arrows) in the nuclear envelope of STIM1^gt/+^ mice as detected by X-gal staining (*n >*4). Scale bar: 500 nm. (**C**) Western blots showing STIM1 expression in the nuclear and cytosolic fractions. (**D** and **E**) STIM1 expression in D84G fibers (*n* >6). Scale bar: 10 μm. (**F**–**H**) Nuclear abnormalities in D84G mice as demonstrated by DAPI staining (blue) (*n =* 7 mice per genotype). (**F** and **G**) Nuclei along the length of the fiber in WT (**F**) and STIM1^+/D84G^ (**G**) mice. Scale bar: 50 μm. (**H**) Quantification of abnormal nuclei (large, clumped, lobular or fragmented) (percentage) in WT and STIM1^+/D84G^ mice. Values are the mean ± SD. *n = 7* independent experiments. *P < 0.05, **P < 0.01, ***P < 0.001, and ****P < 0.0001, by 2-tailed Student’s *t* test (NS, *P >* 0.05). Data are shown as the average number of abnormal nuclei in all fibers (*n =* 48 fibers per genotype). (**I**–**P**) Transmission electron micrographs of nuclei in TA muscles of WT (**I**) and STIM1^+/D84G^ (**J**–**P**) mice (*n >*3). Scale bar: 2 μm. (**J**) D84G nucleus with fragmented micronuclei (red arrow). (**K** and **N**) D84G nuclei with dilated PNS (green arrows). (**K**) D84G nuclei with condensed chromatin (blue arrow). (**L**) Vacuolated nucleus in D84G mice (purple arrow). (**M**) Pale, fading nucleus suggesting karyolysis (black arrow). (**O** and **P**) Vacuolated nuclear membrane (yellow arrows).

Given the changes in muscle mass and altered SR ultrastructure in muscles of STIM1^+/D84G^ mice, we next considered that the mutant D84G STIM1 was not properly targeted in the SR. FDB fibers from WT mice immunostained with STIM1 antibodies and DAPI to label nuclei revealed the presence of STIM1 in the jSR, the lSR near the Z-line, and throughout the nuclear membrane, as previously shown (11). STIM1 is enriched in the nuclear reticulum (NR), which is a specialized nuclear envelope invagination (NEI) that extends from the cytosol into the nucleoplasm (Figure 5A). Confirmation of STIM1 in the nuclear membrane was obtained by TEM of muscles taken from STIM1 reporter mice (STIM1^+/LacZ^ mice), whereby 1 copy of STIM1 was fused to β-galactosidase (4). The STIM1-LacZ appeared as a black precipitate and was clearly expressed in the nuclear envelope (Figure 5B). Additional evidence for STIM1 in the nuclear envelope (NE) was apparent by Western blotting of nuclear fractions isolated from the muscles of WT and STIM1^+/D84G^ mice (Figure 5C). Blotting for histone and GAPDH demonstrated good separation of the cytosolic and nuclear fractions. STIM1-L, a spliced variant of STIM1, was enriched in the nuclear fractions compared with STIM1-S (Figure 5C, upper panel). Both STIM1-S and STIM1-L variants were present in the cytosolic fraction (Figure 5C, lower panel). No differences in the STIM1-L nuclear expression were detected in WT or STIM1^+/D84G^ muscle.

In STIM1^+/D84G^ muscle fibers, we detected STIM1 in the lSR, in a striated pattern typical for the SR (Figure 5, D and E). Nuclear staining in STIM1^+/D84G^ muscle fibers resembled that seen in the WT fibers for many nuclei. However, we observed a number of nuclei with abnormal STIM1 staining, and these altered patterns were associated with a change in nuclear morphology (Figure 5, D and E). STIM1 staining in the NE of STIM1^+/D84G^ muscle fibers often appeared distorted and occupied a much broader region of the NE. Here, STIM1 was detected throughout the NE and can be seen bisecting the nucleus unevenly from what appears to be abnormal NEI, as DAPI (a marker for DNA) was absent from this region. Nuclear morphology varied enormously between WT and STIM1^+/D84G^ fibers, in which STIM1^+/D84G^ ranged from small micronuclei to massive nuclei with multiple lobes (Figure 5, F and G). WT nuclei were always positioned underneath the sarcolemma, whereas D84G nuclei were seen in the middle of fiber, often displacing myofibrils. We quantified nuclear abnormalities in both genotypes and found that STIM1^+/D84G^ mice had fibers with more abnormal nuclei, as depicted by the histogram in Figure 5H showing the distribution percentage of abnormal nuclei and the significant difference in distribution between WT and STIM1^+/D84G^ fibers. The average of all fibers analyzed revealed that approximately 17.4% of nuclei from STIM1^+/D84G^ fibers were clumped, aggregated, misshapen, or broken down. In contrast, only 4.7% of WT nuclei were characterized as abnormal, and these involved 2 nuclei that were indistinguishable but had to be counted for consistency (Figure 5, F–H). These data suggest that the presence of the mutant STIM1 in these fibers compromised the structure and integrity of the NE. Given the changes in nuclear shape of STIM1^+/D84G^ fibers, we used TEM to better assess the nuclear architecture of skeletal muscle (Figure 5, I–P). We found evidence of nuclear rupture and herniation, dense chromatin accumulation, and markedly dilated nuclear invaginations. Importantly, the perinuclear space (PNS) was also found to be dilated for myonuclei from STIM1^+/D84G^ muscles. Finally, we were able demonstrate connections, albeit rare, between the tubular aggregates in the SR and the dilated PNS in some fibers (Figure 5K). These data show that constitutively active STIM1 mutations disrupted nuclear structures, which contribute to the pathogenesis for STIM1 myopathy.

### D84G STIM1 destabilizes the LINC complex and nuclear lamina.

**Figure 6:**
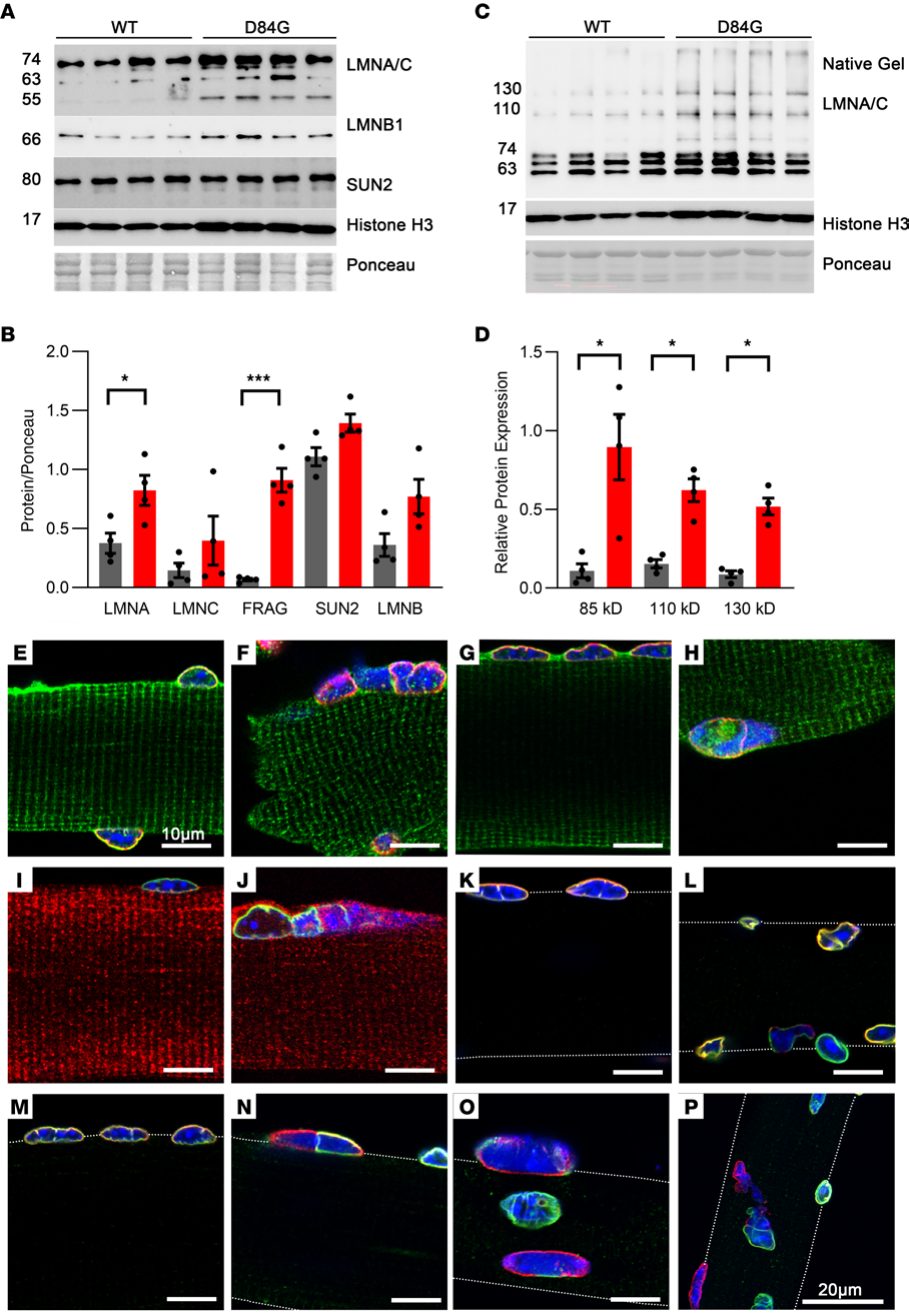
Nuclear membrane proteins in WT and D84G mice. Nuclear extracts from GST muscle were subjected to SDS-PAGE and immunoblotting for WT (*n =* 4) and STIM1^+/D84G^ (*n =* 4) male mice. (**A**) Immunoblotting for LMNA/C, LMNB1, SUN2, and histone H3. LMNA/C blots are represented by isoforms (60–80 kDa) and smaller fragments (55 kDa). (**B** and **D**) Quantification of protein expression in WT (black) and STIM1^+/D84G^ (gray) muscle tissue from **A**, showing LMNA and 54 kDa fragment highly expressed in STIM1^+/D84G^. (**C**) Quantification of native protein expression in WT and STIM1^+/D84G^ tissue, with more LMNA/C aggregations in STIM1^+/D84G^ samples. Values are the mean ± SD. *P < 0.01 and ***P < 0.001, by 2-tailed Student’s *t* test. (**E**–**P**) Localization of nuclear membrane proteins in FDB fibers by fluorescence immunohistochemistry (*n =* 3–5 mice). (**E** and **F**) STIM1 (green) and LMNA/C (red) in (**E**) WT and (**F**) STIM1^+/D84G^ mice. (**G** and **H**) STIM1 (green) and SUN2 (red) in (**G**) WT and (**H**) STIM1^+/D84G^ muscle. (**I** and **J**) LMNB1 (green) and STIM1 (red) in (**I**) WT and (**J**) STIM1^+/D84G^ muscle. (**K** and **L**) LMNB1 (green) and SUN2 (red) in (**K**) WT and (**L**) STIM1^+/D84G^ muscle. (**M**–**P**) LMNB1 (green) and LMNA/C (red) in (**M**) WT and (**N**–**P**) STIM1^+/D84G^ muscle. (**E**–**O**) Confocal images taken at ×40 magnification with ×5 zoom. Scale bars: 10 μm. (**P**) Maximum image projection of 11 confocal images taken at 2 μm intervals through the fiber at ×40 magnification with ×3 zoom. Scale bars: 20 μm. *n =* 3–5 independent experiments.

We next considered a recently published database by Gu et al., in which proximity BioID proteomics was performed using STIM1 as one of many baits in HEK293 cells (23). Of the 173 proteins identified to be in close proximity to STIM1 with this assay are proteins located at the ER, golgi, and NE. We used NIH DAVID Bioinformatics Resources (https://david.ncifcrf.gov/home.jsp) to identify signaling pathways and disease terms associated with this group of proteins and found that terms including nuclear cytosolic transport and EDMD were significantly linked to STIM1 proximity (Supplemental Table 1) (23, 24). We therefore hypothesized that components of the nuclear lamina, which is the meshwork of intermediate filaments that form part of the NE, would be altered in the muscle fibers of STIM1^+/D84G^ mice (25). LMNB1 and SUN2 expression was unchanged in nuclear extracts of STIM1^+/D84G^ muscle (Figure 6A). We detected LMNA/C as 2 bands on immunoblots for WT muscle, consistent with the LMNA (74 kDa) and LMNC (63 kDa) isoforms. An increase in LMNA isoform levels was apparent in the nuclear extracts of STIM1^+/D84G^ mice, whereas LMNC levels did not differ by genotype. On further examination, we detected additional species with the LMNA/C antibody (55 kDa), indicating a most likely cleavage of LMNC in the STIM1^+/D84G^ nuclear extracts (26) (Figure 6, A and B). These LMNC fragments migrated as high-molecular-weight multimers under nonreducing PAGE conditions (85–150 kDa) (Figure 6, C and D). In addition, this 55 kDa LMNA/C fragment was detected in greater amounts in the soluble cytosolic fraction of the STIM1^+/D84G^ muscles, consistent with LMNC damage (Supplemental Figure 2A). To further test whether STIM1 D84G disrupted LMNA/C and caused nuclear export of LMNA/C, we compared LMNA-GFP localization in HEK-293 cells expressing either WT STIM1 or STIM1 D84G. LMNA-GFP was detected outside the nucleus to a greater extent in cells expressing STIM1 D84G (Supplemental Figure 2C). We interpret these data as evidence that the D84G-mutant STIM1 in the NE destabilized the nuclear lamina and LMNA/C filamentous network (Supplemental Figure 3A).

Costaining FDB fibers for STIM1 along with LMNA/C (red) (Figure 6, E and F), SUN2 (red) (Figure 6, G and H), or LMNB1 (green) (Figure 6, I and J) demonstrated colocalization of LMNA/C, LMNB1, and SUN2 with STIM1 in WT nuclear membranes. In the majority of nuclei in STIM1^+/D84G^ fibers, we found that STIM1 also colocalized with LMNA/C, SUN2, and LMNB1. However, there were numerous nuclei with marked disorganization of the nuclear lamina in STIM1^+/D84G^ fibers. Furthermore, there was an absence of lamin A/C (red) staining in a number of dysmorphic nuclei (Figure 6F). SUN2 (red) was generally present in all nuclear membranes but downregulated in some nuclei with disrupted morphology (Figure 6, G and H). LMNB1 (green) was, again, absent in a number of nuclei of STIM1^+/D84G^ muscles and was notably absent from micronuclei (Figure 6, I, J, and P). To better understand the differences in nuclear lamina in D84G fibers, we costained for LMNA/C (red) and SUN2 (red) with LMNB1 (green). In contrast to WT nuclei, where there was good colocalization for LMNB1 and SUN2 in muscle nuclei, LMNB1 (green) staining was often lost in portions of misshapen nuclei from the STIM1^+/D84G^ fibers, and SUN2 was evident in all nuclei but was downregulated in some regions of the NE from STIM1^+/D84G^ nuclei (Figure 6, K and L). Similarly, LMNA/C (red) was often detected independent of the LMNB1 (green) in the nuclei of STIM1^+/D84G^ fibers but showed strong colocalization in WT nuclei (Figure 6, M–P). These data are consistent with the notion that the nuclear lamina was damaged in nuclei expressing D84G-mutant STIM1, as evidenced by the altered spatial localization of nuclear lamina and the LINC complex.

### LMNA-dependent gene expression is altered in STIM1^+/D84G^ muscle.

**Figure 7:**
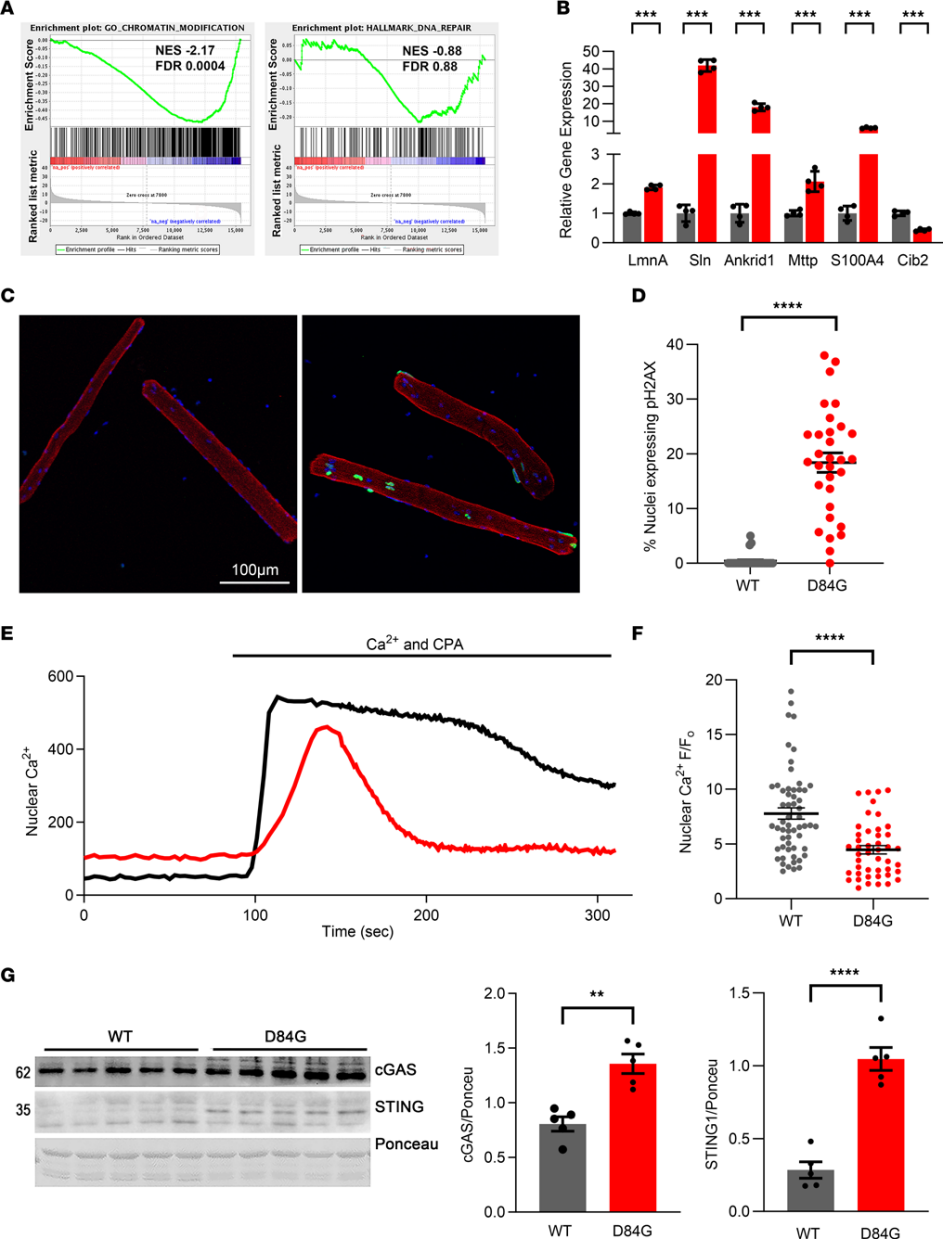
Muscle fibers from STIM1^+/D84G^ mice exhibit nuclear dysfunction. (**A**) GSEA of WT versus STIM1^+/D84G^ RNA-Seq. The FDR *q* value and the *P* value are shown for each plot. Negative enrichment of gene sets involved in DNA repair and chromatin organization are shown. (**B**) LMNA target gene expression in WT and STIM1^+/D84G^ muscle. RNA-Seq data from WT and STIM1^+/D84G^ muscle were mined for genes known to be differentially expressed in LMNA-KO muscle, as described previously (27, 28). Quantification of results (mean ± SEM; *n* =4 animals for each genotype). ***P < 0.01, by 2-tailed Student’s *t* test only for STIM1^+/D84G^ versus WT. (**C**) FBD fibers from WT and D84G mice were fixed and immunostained with the γ-H2A.X antibody (green) to detect phosphorylated histone H2A levels, a marker of DNA damage. Nuclei are colabeled with DAPI (blue). Contours of the fiber are shown by phalloidin to label actin (red). Results are representative of 3 mice of each genotype. Scale bar: 100 μm. (**D**) Quantification of nuclei expressing phosphorylated histone H2A in FBD fibers from WT and D84G mice. (**E** and **F**) WT and D84G STIM1 myoblasts were transfected with GCaMP6mXn (gCAMP), a genetically encoded Ca^2+^ indicator. The SERCA inhibitor CPA (30 μM) was used to evoke Ca^2+^ transients, and then Ca^2+^ was added back to reestablish [Ca^2+^]_N_. Comparison of the nuclear Ca^2+^ content was determined as the F/F_0_ (F_0_ is the average lowest level in no-Ca^2+^ CPA, and F is the difference of the peak reading with readmitted Ca^2+^ and F_0_). Results are representative of WT (*n =* 57) and D84G mutant (*n =* 46) myoblasts. Imaging was performed on 5 separate days. Results are the mean ± SEM. ****P < 0.001, by 2-tailed Student’s *t* test for STIM1^+/D84G^ versus WT STIM1 transfected myoblasts. (**G**) Nuclear lysates from WT (*n =* 5) and STIM1^+/D84G^ (*n =* 5) mouse muscle were prepared and immunoblotted for cGAS and STING with specific antibodies. Ponceau staining was used as the loading control. Quantification of results (mean ± SEM) is shown on the right. **P < 0.01 and ****P < 0.001, by 2-tailed Student’s *t* test for STIM1^+/D84G^ versus WT.

To this point, our findings showed that nuclei of STIM1^+/D84G^ muscles were distorted and that the nuclear lamina was damaged, features that resembled those of a NE subjected to excessive mechanical forces or as part of the pathology seen in laminopathies. We hypothesized that changes in the nuclear lamina of STIM1^+/D84G^ muscles would interrupt LMNA-chromatin interactions, leading to the expression of genes that are otherwise silenced, as has been described in LMNA-KO muscles (27, 28). Consistent with this idea, gene set enrichment analysis (GSEA) of our RNA-Seq data from STIM1^+/D84G^ muscles revealed negative enrichment of genes regulating DNA repair and chromatin modification (Figure 7A). To confirm this idea, we quantified a set of genes known to be upregulated in the LMNA-KO muscles and found profound changes in these mRNA levels in the muscles of STIM1^+/D84G^ mice, including for *Sln* (40-fold), ankryin domain repeat 1 (*Ankrd1*) (18-fold), microsomal triglyceride transfer protein (*Mttp*) (2-fold), Ca^2+^ and integrin binding proteins (*Cib2*) (0.5-fold reduction), and S100 Ca^2+^ binding protein A4 (*S100A4*) (9-fold increase) (Figure 7B). Collectively, these data indicate that D84G STIM1 in the NE probably led to an alteration in the lamina-associated chromatin structure, which may account for the large number of DEGs in STIM1^+/D84G^ muscles.

### DNA damage in the STIM1^+/D84G^ muscles.

The foregoing observations that muscle fibers expressing the STIM1 D84G mutant exhibited damage to nuclear lamina and altered LMNA/C gene expression raised the possibility that DNA damage may accumulate in the nuclei of STIM1^+/D84G^ muscle and thus represent an important mechanism underlying reduced muscle growth and weakness. Genomic instability can be detected at sites of double-stranded breaks in DNA in muscle nuclei, as an increase in phosphorylation of histone H2A.X. DNA damage was detected by the presence of γH2A.X^+^ myonuclei in STIM1^+/D84G^ muscle fibers. While no γH2AX^+^ fibers were detected in the WT muscles, 20% of myonuclei from STIM1^+/D84G^ muscles exhibited a significant number of γH2A.X^+^ myonuclei, indicating that D84G STIM1 associated with nuclear lamina damage and caused DNA damage (Figure 7, C and D, n *=* 3 mice, *P <* 0.0001). The γH2AX^+^ myonuclei were most often the misshapen nuclei, consistent with loss NE integrity.

### D84G STIM1 reduces nuclear Ca^2+^.

We next wanted to determine whether the D84G mutation in STIM1 influences the nuclear Ca^2+^ concentration ([Ca^2+^]_N_). In resting muscle cells, [Ca^2+^]_N_ is maintained by tonic leak of Ca^2+^ from IP3R channels and Ca^2+^ efflux by SERCA1 (29–31). In contrast, the nuclear pores (NPC) act as passive conduits for Ca^2+^ during cell stimulation and determine the amplitude of [Ca^2+^]_N_ (32, 33). The genetically encoded Ca^2+^ sensor gCAMP6f can be targeted to the nucleus by the addition of a SV40 nuclear localization sequence (34). We induced the expression of n-gCAMP6 in myoblasts expressing either WT or D84G STIM1. Myoblasts were subjected to Ca^2+^ store depletion by application of the SERCA inhibitor CPA in the absence of external Ca^2+^, a protocol known to activate STIM1. Upon readdition of Ca^2+^, [Ca^2+^]_N_ increased for both WT and STIM1 D84G myoblasts, as Ca^2+^ entered the nucleus by the actions of the NPC. Under these conditions, the amplitude of [Ca^2+^]_N_ was significantly reduced in the D84G STIM1–expressing muscle cells compared with WT (Figure 7, E and F). These findings are consistent with the idea that D84G STIM1 influences Ca^2+^ flux across the NE and is most likely mediated by the NPC. Consistent with this idea, we found a significant reduction in mRNA transcript reads for several components of the NPC in the RNA-Seq data set from STIM1^+/D84G^ muscles (Supplemental Figure 3B).

Additional evidence for DNA damage in the D84G-mutant fibers included upregulation of the enzyme cyclic GMP-AMP synthase (cGAS), which is a DNA sensor (Figure 7G). Similarly, Western blotting for stimulator of IFN genes (STING1) was increased in muscles of STIM1^+/D84G^ mice (Figure 7G). STING is activated by cytosolic DNA and cGAS, thereby contributing to DNA damage signaling and activation of the sterile inflammation cascade. Together, these data show that the presence of D84G STIM1 in the NE creates proteostatic stress and DNA damage, which impairs muscle growth and performance, as seen in the STIM1^+/D84G^ mice.

## Discussion

In the present work, we provide evidence that a gain-of-function mutation in the STIM1 gene is sufficient to induce a progressive myopathy accompanied by minor changes in Ca^2+^ signaling and drastic changes in nuclear structure and function. Specifically, we show that mice harboring a single nucleotide change in STIM1 (D84G) displayed (a) constitutive Ca^2+^ entry across the sarcolemma of muscle fibers, (b) reduced muscle mass and weakness consistent with sarcopenia, and (c) disruption of the NE resulting in altered nuclear architecture, gene expression, and DNA damage. Overall, these studies establish that STIM1^+/D84G^ mice replicate the clinical aspects of weakness and reduced muscle mass that occur in humans and offer insight into the function of STIM1 in the NE and highlight how STIM1 links mechanical signals with gene expression, DNA damage, and muscle growth.

In skeletal muscle, STIM1 localizes to specialized SR domains, where it interacts with different target proteins to carry out distinct cellular functions. For example, STIM1 is present in the membrane of the SR terminal cisternae, where it activates Orai1 channels in the adjacent T-tubule membrane (4). Far less is known about the role of STIM1 in the NE of muscle cells (12). We detected STIM1 in the NE and throughout the invaginations of the NE called the nuclear reticulum, as was previously shown (35). In an attempt to understand the function of STIM1 in the NE, we characterized the nuclear phenotype of STIM1^+/D84G^ mice. TEM of muscles from STIM1^+/D84G^ mice demonstrated expansion of the space between the ONM and INM PNS as well as altered nuclear morphology. These findings are reminiscent of prior studies of cells depleted of SUN proteins that exhibited an enlarged PNS due to loss of key connections between SUN1 and SYNE1 in the ONM (36). In SUN1/SUN2-KO mice, defects in myonuclei anchorage results from the failure to retain nesprin 1 at the ONM. The mutant STIM1 present in the ONM may also disrupt key components of the LINC complex and thereby activate nuclear stress signaling. Whether STIM1 connects the NE to the cytoskeleton, where it may integrate mechanical signaling, requires further investigation. In fact, we recently demonstrated that STIM1 interacts with desmin, a muscle-specific cytosolic intermediate filament. Given that desmin is known to interact with nesprin1 or nuclear envelope spectrin repeat protein 1 (SYNE1) to stabilize nuclear positioning and maintain the nuclear architecture of muscles, we propose that STIM1 and desmin establish another important SR domain in muscle, in this case with the NE, where it is involved in mechanotransduction for the muscle (37, 38).

Disruption of the folding of the EF hand for STIM1 by the D84G mutation eliminates the Ca^2+^ sensor function of STIM1, leading to constitutive SOCE in muscle fibers. Importantly, muscle fibers adapt to the constitutive SOCE, as there was no change in Ca^2+^ stores, resting Ca^2+^, or EFS-evoked Ca^2+^ transients. The upregulation of SLN and CASQ expression in STIM1^+/D84G^ muscle provides adaptation to the augmented expression of SOCE by enhancing Ca^2+^ storage and limiting SERCA1 refilling. In contrast, our studies show that cytosolic transfer of Ca^2+^ into the nucleus by the NPC was reduced when D84G STIM1 was expressed in myoblasts (39, 40). The consequence of lower [Ca^2+^]_N_ in STIM1^+/D84G^ myoblasts was likely due to altered mechanical properties of the nucleus. These data are consistent with recent studies showing that mechanical stress disrupts the link between LMNA/C, lamina-associated chromatin, and nuclear Ca^2+^ levels that subsequently influence chromatin structure, chromatin accessibility, and gene expression (41). Our RNA-Seq data from muscles of 6-month-old STIM1^+/D84G^ mice demonstrated a very significant, approximately 10% increase in DEGs. These changes likely reflect disruption of chromatin contacts with the lamina, as indicated by the GO analysis and the enhanced expression of LMNA/C-regulated genes. The nuclear periphery is often aligned with condensed chromatin that harbors genes that are transcriptionally silent (42). We therefore interpret our studies as evidence that nuclei of STIM1^+/D84G^ muscle resemble nuclei in WT muscle subjected to excessive stretch, where the heterochromatin structure is modified and ER/nuclear Ca^2+^ levels are altered, resulting in DNA damage (43). This work therefore establishes a previously unappreciated role for STIM1 in regulating NE dynamics and a possible role in mechanotransduction and gene expression.

The clinical syndrome of muscle atrophy and weakness develops in humans as a result of gain-of-function mutations in the *STIM1* and *ORAI1* genes. Pathologic biopsy specimens and MRI imaging demonstrate tubular aggregates in muscles of these patients has led to the assertion that constitutive SOCE causes tubular aggregates (44). Studies to date have failed to show tubular aggregates in the muscles of STIM1 and ORAI1 mouse models despite the presence of constitutive SOCE, reduced muscle growth, and muscle weakness (5–9, 16, 44–52). In contrast, tubular aggregates are often detected in the muscles of aging WT mice, in which the regulation of SOCE is probably altered. Although changes in SOCE have been proposed to occur in aging muscle, it remains to be determined if STIM1-dependent Ca^2+^ signaling is coincidently reduced in the aging muscle (53, 54). The results of these studies question whether STIM1 and Orai1 mutations are the cause of tubular aggregate formation. Based on our work with this STIM1-mutant mouse model, we propose that STIM1 senses mechanical stress as well as Ca^2+^ stores content in the NE and nuclear lamina. STIM1 can then influence nuclear Ca^2+^ flux through the NPC, limit LMNA-chromatin changes, and prevent DNA damage. This work establishes a previously unrecognized role for STIM1 signaling in the nuclear dynamics that is likely distinct from SOCE. Finally, it is important to note the relevance of this signaling pathway to aging and that STIM1 has a physiologic role in sinus node pacemaking and arrhythmias, immune responsiveness, and muscle metabolism — all of which are perturbed with aging. The implication that STIM1 is a potential therapeutic target for aging therefore requires further study.

## Methods

### Sex as a biological variable

Our study examined male and female animals, and similar findings are reported for both sexes.

### Animals

*Stim1^+/D84G^* mice were generated as described previously (7). Sequencing of this mouse line showed an A-to-G transition at nucleotide 444 in exon 2 of *Stim1* (NM_009287), resulting in an amino acid exchange in the EF hand motif at position 84 (Asp84Gly or D84G). Age- and sex-matched C57BL/6 mice were used as WT controls.

#### Isolation of FDB fibers.

FDB muscles were carefully dissected from the foot and placed into DMEM with 0.5–1 mg/mL collagenase A (Roche, 10103578001). Fibers were digested at 37°C overnight, with rocking, before isolation by gentle trituration in DMEM using a glass pipette. Once isolated, fibers were plated on 35 mm glass-bottomed dishes coated with 20 μg/mL laminin (MilliporeSigma, L2020).

### Nuclear enrichment

Frozen skeletal muscle tissue was powdered with liquid nitrogen and resuspended in SMT buffer (0.2% NP40, 250 mM sucrose, 50 mM Tris HCl, 5 mM MgCl2, pH 7.6, with protease inhibitors) for homogenization and centrifuged at 1,000*g*, and the pellets were collected. The nuclear pellet was washed with SMT buffer 3 times and resuspended in NET buffer (20 mM Tris.Cl, 150 mM NaCl, 1.5 mM MgCl_2_, 1% SDS, and 5% glycerol, pH 7.6, with a protease inhibitor), vortexed for 15 seconds, passed through a 20 gauge needle, and then centrifuged at 11,000*g* for 15 minutes to collect the nuclear lysate.

### Immunoblotting

Standard protocols were used for immunoblot analysis. Blots were incubated overnight at 4°C with primary antibodies. Antibodies against STIM1 (MilliporeSigma, catalog 6197), SERCA1a (Thermo Fisher Scientific, ma3912 mouse), RyR (Thermo Fisher Scientific, 3925), CASQ (Thermo Fisher Scientific, 3913 mouse), SLN (MilliporeSigma, ABT13), lamin A/C (Cell Signaling Technology, antibody 4C11, mouse mAb), SUN2 (MilliporeSigma, MABT880), cGAS (Cell Signaling Technology, 31695), STING1 (Cell Signaling Technology, 50494), histone H3 (Cell Signaling Technology, 9715s), and GAPDH (MilliporeSigma, G9545) were used. Signals were detected using an HRP-conjugated secondary antibody and the Pierce Super Signal Western Dual Extended Duration Substrate kit (Thermo Fisher Scientific, 34075) and were quantified using the ChemiDocMP Imaging System (Bio-Rad).

### Histology

Fibers were fixed in 4% PFA for 5 minutes or in acetone for myosin staining, washed in PBS, and blocked in 10% heat-inactivated goats serum (HINGS). Fibers were incubated with a primary antibody overnight at room temperature in fiber antibody solution (PBS, 2% HINGS, and 0.3% Triton). The primary antibodies used are listed in Supplemental Table 2. After washing, fibers were incubated with a secondary antibody for 1 hour (molecular probes, Alexa Fluor series), washed, and then mounted in VECTASHIELD. Nuclei in whole fibers were stained for 5 minutes with 10 μg/mL DAPI. Staining was analyzed on a Zeiss 510 confocal microscope. For staining of cryosections on slides, a similar protocol was used, but the antibody solution was 1% HINGS with 0.1% Triton.

### Electron microscopy

For ultrastructural localization of STIM1-LacZ by TEM, FDB muscles were fixed in situ for 5 minutes in 2% PFA 0.2% gluteraldehyde in PBS. Nodes were then dissected and stained for LacZ as described in Stiber et al. (4). Tissue was postfixed in 2% PFA and 2% glutaraldehyde in 0.1 M phosphate buffer, pH 7.4, and then fixed in 1% osmium tetraoxide and stained en bloc with 1% uranyl acetate. The tissue was then dehydrated in a graded ethanol series, taken through a series of Spurr resin/ethanol washes, and embedded in Spurr resin. Thin sections were cut at 70 nm, mounted on copper grids and counterstained with 2% uranyl acetate and lead citrate. Grids were viewed and photographed using a FEI Tecnai G2 Twin transmission electron microscope. Semi-thin sections were cut at 1 μm and then mounted on slides and dried on a hotplate. They were stained with 1% toluidine blue and 2% borate in distilled water with the hotplate still on. Excess stain was removed with distilled water and dried. Slides were cover-slipped in CytoSeal 60.

### Calcium imaging of FDB myofibers

#### Manganese quench.

Fibers were loaded with 1 μg/mL Fura-2AM for 30 minutes in Tyrode’s solution (121 mM NaCl, 5 mM KCl, 1.8 mM CaCl_2_, 500 μM MgCl_2_, 400 μM NaH_2_PO_4_, 100 μM EDTA, 5.5 mM glucose, and 24 mM NaHCO_3_). Solutions were bubbled with oxygen throughout. Fura-2AM was removed by perfusing with Tyrode’s solution with 50 μM *N*-benzyl-*p*-toluene sulfonamide (BTS) to prevent motion artifact. During this period of time, healthy fibers were selected by applying a single stimulus for 100 ms (100 mA) at 50 Hz. Fibers were imaged for 3 minutes in Tyrode’s solution with Ca^2+^ to determine the basal level of Fura-2. The Ca^2+^ was subsequently replaced with 1.8 mM Mn by perfusion for 5 minutes at 1 mL/minute. Fibers were then stimulated with 20 trains at 50 Hz for a 1-second duration every 5 seconds using an Acupulser 310 (World Precision Instruments) with an A385 Stimulus Isolator (World Precision Instruments) was used. A stimulation dish was purchased from World Precision Instruments (RC-37FS). The florescence signal was monitored throughout the experiment using an excitation filter at 360 nm and an emission filter at 510 nm. Images were collected every 2 seconds on a Nikon TE2000 inverted microscope equipped with a Photometrics CoolSNAP camera and a Lambda DG-4 rapid filter changer (Sutter). Image acquisition was controlled using Metafluor/Metamorph software (Molecular Devices). Images were acquired using a 40× S Plan 1.3 numerical aperture (NA) objective lens.

#### Basal calcium.

Fibers were loaded with 1 μg/mL Fura-2AM for 30 minutes in oxygenated imaging solution (120 mM NaCl, 5 mM KCl, 2 mM CaCl_2_, 1 mM MgCl_2_, 25 mM NaHCO_3_, 0.5 mM NaH_2_PO_4_, and 10 mM glucose). Fura-2 was washed out of the dish for 5 minutes, and fibers were selected for viability. Ca_2+_ signals were measured by alternate excitement at 340 nm and 380 nm and emission at 510 nm. Acquisition was every second on a PCO Edge 5.5 camera, core LED 340 Fura light source. Image acquisition was controlled using Metafluor/Metamorph software (Molecular Devices). Images were acquired using a 40× S Plan 1.3 NA objective lens.

#### Fiber stimulation.

Fibers were loaded with 2 μg/mL Fura-4F AM for 1 hour in oxygenated imaging solution. Fura-4F AM was removed by perfusion with imaging solution with BTS for 5 minutes. Fibers were selected for viability and then subjected to a stimulation protocol while being perfused with oxygenated imaging solution. A stimulus response curve was generated by applying stimuli ranging from 100 milliseconds to 2 seconds every 45 seconds. After this, trains of stimuli were applied with 25 stimuli of 500 milliseconds every 5 seconds first, and then 25 stimuli of 2 seconds every 5 seconds. Ca^2+^ signals were measured by alternate excitement at 340 nm and 380 nm and emission at 510 nm. Acquisition was done every 180 milliseconds at 340 nm and 80 milliseconds at 380 nm on a PCO Edge 5.5 camera, core LED 340 Fura light source. The camera and software used are as described for basal calcium measurements.

#### Store depletion.

Fibers were loaded with 2 μg/mL Fura-4F AM for 1 hour in oxygenated imaging solution. Fura-4F AM was removed by perfusion with imaging solution with BTS for 5 minutes. Fibers were selected for viability, and basal calcium levels were recorded. Fibers were then perfused with imaging solution with 0 calcium for 3 minutes. Stores were depleted using a solution with 0 calcium, 30 μM CPA, and 10 mM caffeine.

#### Nuclear Ca^2+^ measurements.

To measure nuclear Ca^2+^, plasmids encoding WT and D84G STIM1 were transfected into C2C12 cells with the genetically encoded Ca^2+^ indicator pEGFP-N1-GCaMP6m-Xn (a gift from Xiaodong Liu, Addgene plasmid no. 118976; http://n2t.net/addgene:118976; RRID: Addgene_118976) targeted to the nucleus. Wide-field epifluorescence was recorded for the GFP signal. Myoblasts were perfused with HBSS (Gibco, Thermo Fisher Scientific, 14175-095) with 1 mM Mg^2+^ for the SOCE assay, including no Ca^2+^ solution and no Ca^2+^ with cyclopiazonic acid (30 μM) to deplete stores, and then 2 mM Ca^2+^ with cyclopiazonic acid (CPA) were added back. Changes in nuclear Ca^2+^ were determined from the peak of the signal after readdition of Ca^2+^, normalized to the average lowest level in no-Ca^2+^ CPA.

### Behavioral studies

#### Open-field activity.

Open-field activity was monitored using the VersaMax Animal Activity Monitoring System. Mice were tested in individual chambers for a total of 15 minutes before and immediately after exercise. Every 5 minutes of ambulation, the average total distance and time spent in movement as well as vertical activity were determined, and standard errors were calculated. Cumulative measures for every 5-minute interval were charted. Mice were exercised with an adjustable, variable-speed belt treadmill from AccuPacer as previously described (33).

#### Grip strength.

Measurements of grip strength were done for each individual mouse using a San Diego Instruments animal grip strength system. Grip strength maximum force (N) readings were taken in triplicate, and hind paw strength was calculated indirectly after measurements of front paw and whole-body strength.

#### Involuntary running.

Mice were trained to run on the treadmill for 3 days at a speed of 5 meters/minute for 10 minutes. When they stopped running, the mice were tapped lightly to encourage them to restart. For the experimental run, mice started at a speed of 5 m/min for 2 minutes, and the speed was increased by 1 m/min until the mice were no longer able to run. The endpoint was taken when mice were tapped 3 times and failed to restart.

### Real-time PCR analysis and RNA-Seq

RNA was isolated using a TRIzol extraction protocol followed by clean-up using an RNeasy Mini Kit (QIAGEN). Total mRNA was reversed transcribed into cDNA using the Applied Biosystems High-Capacity mRNA to cDNA kit. cDNA was amplified using TaqMan Gene Expression Master Mix and TaqMan Gene Expression Assays (Thermo Fisher Scientific). The following TaqMan Primers from Thermo Fisher Scientific were used: BIP/HSPA5, mm00517691; PIDA4, mm00437958; SDF21L, mm00452079; GADD45, 45 mm00432079; myogenin, mm00446194; Pax7, mm01354484; myostatin, mm01254459; crystAB, mm00515567; GAPDH, mm99999915; and 18S, Hs99999901.

The Sequencing and Genomics Technologies Core (SGT) at Duke University performed mRNA-Seq. The SGT used the Kapa Stranded mRNA Kit from Roche (code: KK8421) to enrich for mRNA from total RNA and to reverse transcribe mRNA into cDNA to build sequencing libraries. Libraries were pooled to an equimolar concentration and sequenced on the NovaSeq 6000 SP flow cell to produce 50 bp paired-end reads.

### Statistics

Data are presented as the mean ± SEM. Two-tailed Student’s *t* tests were used to calculate *P* values. Two-way ANOVAs were used to compare data sets with multiple time points. A *P* value of less than 0.05 was considered statistically significant.

### Study approval

All mice were maintained in pathogen-free barrier facilities at Duke University and were used in accordance with protocols approved by the Division of Laboratory Animal Resources and the IACUC of Duke University.

### Data availability

The data supporting this study are available from the corresponding author upon reasonable request. Values for all data points in graphs are reported in the Supporting Data Values file. Raw RNA-Seq data generated in this study have been deposited in the Gene Expression Omnibus (GEO) database (GSE261935).

## Supplementary Material

Supplemental data

Supporting data values

## Version 1 02/01/2024

In-Press Preview

## Version 2 04/01/2024

Electronic publication
