# Supplementary material for: The D84G mutation in STIM1 causes nuclear envelope dysfunction and myopathy in mice
Source: J Clin Invest. 2024 Feb 1;134(7):e170317. doi: 10.1172/JCI170317 (PMC10977986; doi:10.1172/JCI170317)
Supplement: Supplemental data [file jci-134-170317-s179.pdf]

Supplemental Figure 1

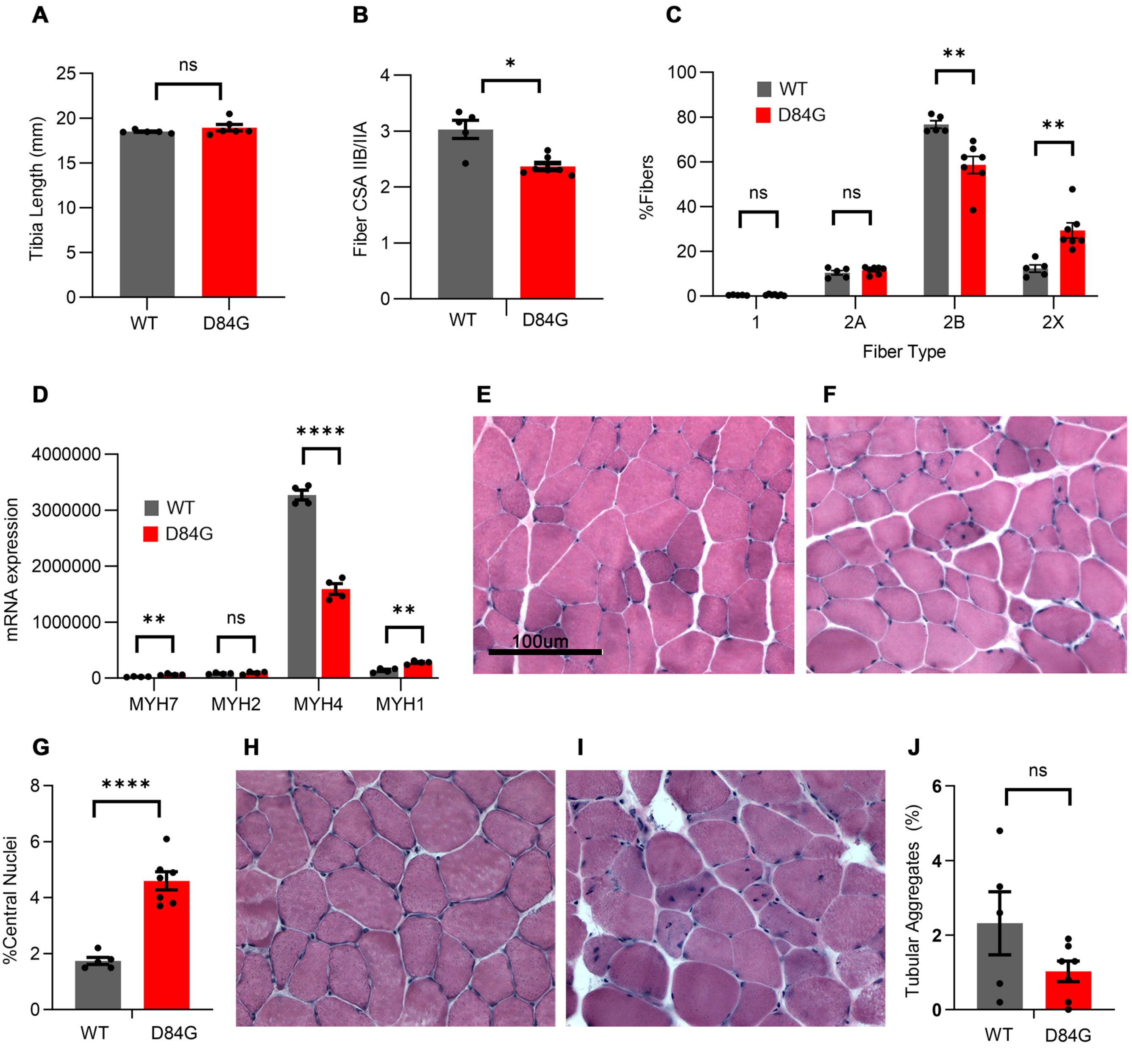

Supplemental Figure 2

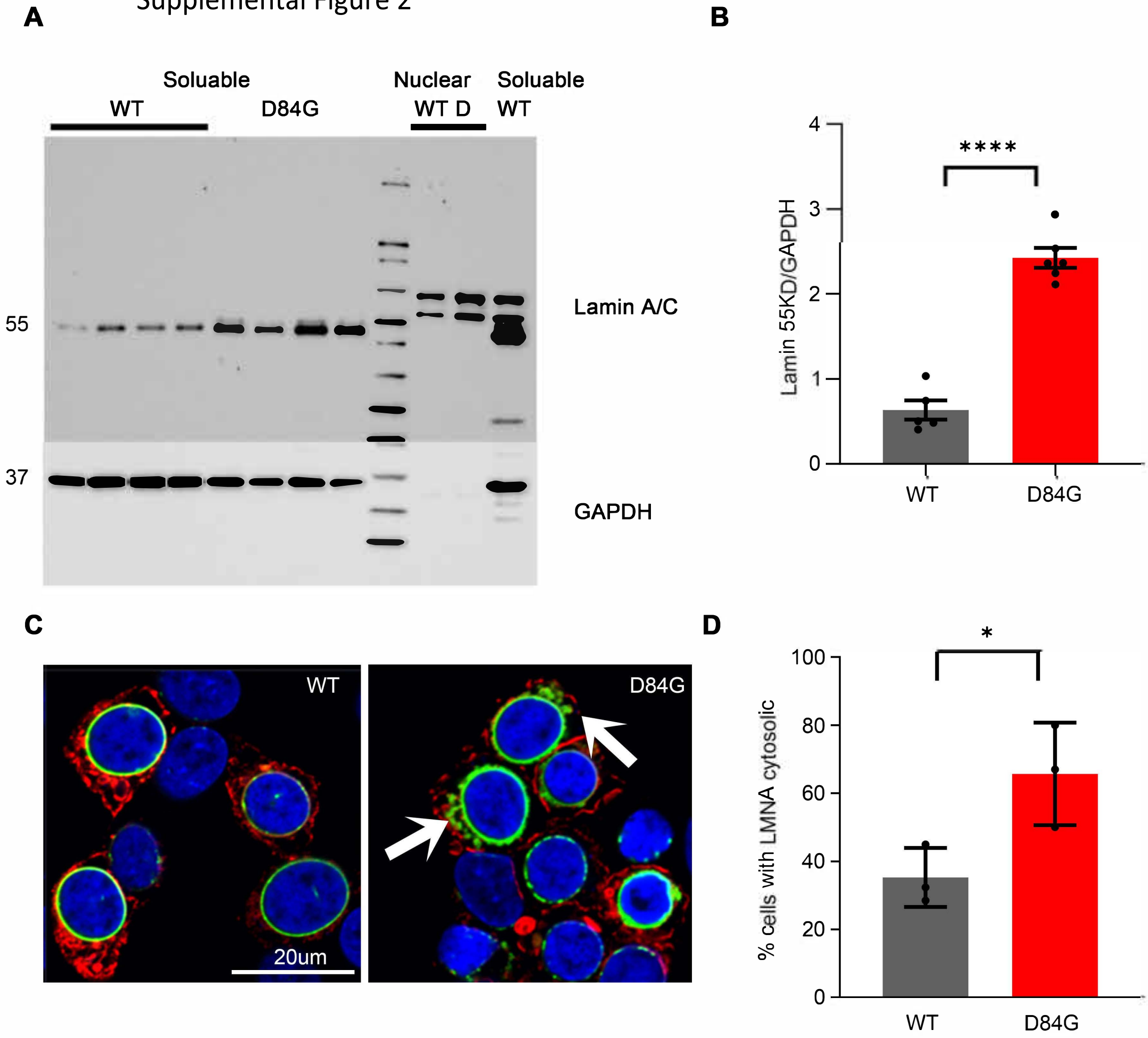

Supplemental Figure 3

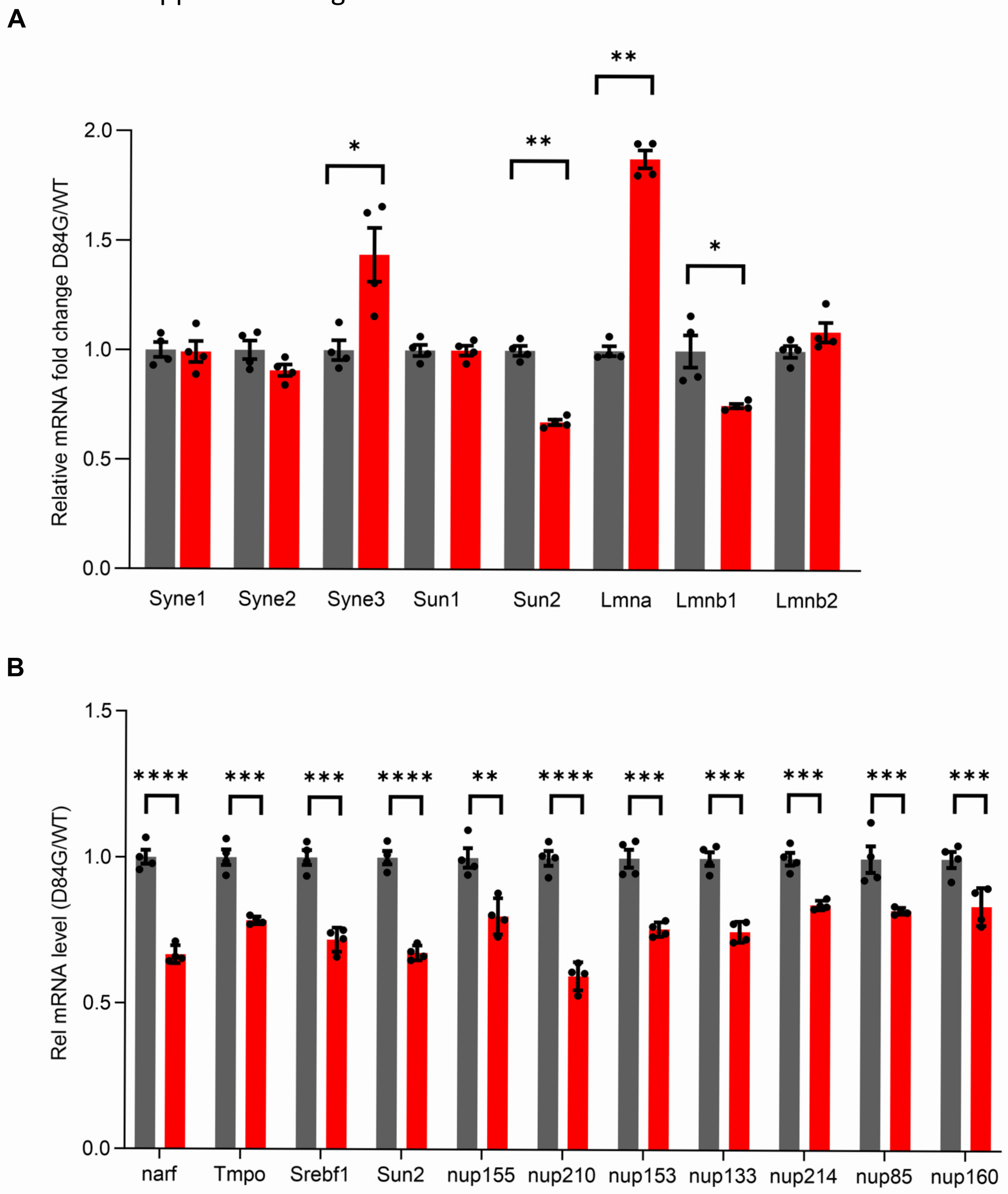

Supplemental Table 1

NIH DAVID Bioinformatics Resources analysis of 173 the STIM1 protein interactions found in the biotinylation map done by Go et al (2021).

Broad Pathways (KEGG or WIKI)

Pathway	Proteins	P- value	Function
Protein Processing in the ER (KEGG PATHWAY)	BCAP31, DNAJC1, SEC24A, SEC24B, SEC63, STT3B, UBXN4, UBXN8, ATF6, CANX, CKAP4, EIF2AK3, LMAN1, PREB, RRBP1, SSR1, UBE2J1	8.0E-13	Protein export, anchoring ribosomes, ER stress response, and degrading misfolded proteins.
SNARE interactions in vesicular transport (KEGG PATHWAY)	SEC22B, YKT6, SNAP29, STX18, STX5, USE1	8.5E-6	Transport vesicles between the ER and Golgi body.
Nucleocytoplasmic transport (KEGG PATHWAY)	NSC1, SENP2, UPF1, NUP107, NUP133, NUP155, NUP98	3.3E-4	Rings of the pores. Transient signal for mRNA transport.
Steroid biosynthesis (KEGG PATHWAY)	DHCR24, NSDHL, LBR, SOAT1	6.5E-4	Cholesterol synthesis and cholesteryl ester synthesis.
RHOG GTPase cycle (REACTOME PATHWAY)	LEMD3, ARHGAP1, VAPB, VANGL1, VRK2, YKT6, ANKLE2, EMD, ESYT1, ITGB1, KTN1, LBR, LMAN1, PGRMC2, STBD1, STX5, TMPO	1.5E-17	Cytoskeleton regulation for cell polarity, migration, and adhesion.
RHOA GTPase cycle (REACTOME PATHWAY)	BCAP31, DDRGK1, FAF2, ARHGAP1, VAPB, VANGL1, YKT6, ACBD5, HMOX2, KTN1, LBR, LMAN1, PGRMC2, SCFD1, STBD1, STX5, TEX2, TMPO	8.4E-14	Cytoskeleton and cell contractility regulation. Effectors in the cytoplasm and ER.<br>In skeletal muscle the RhoA pathway is responsible for muscle differentiation and regeneration (Rodriguez-Fdez and Bustelo 2021).

Cell Cycle (REACTOME PATHWAY)	LEMD3, NDC1, VRK2, ANKLE2, CHMP7, EMD, GORASP2, LBR, LMNB1, MAPRE1, NUP107, NUP133, NUP155, NUP98, PLK1, PKMYT1, SIRT2, SYN1, SYNE2, TMPO, ZW10	1.7E-5	Involved in prophase, metaphase, and anaphase of mitosis.

Disease pathways

Pathway	Proteins	% of Total Pathway Proteins	P - value	Function
Amyotrophic lateral sclerosis (KEGG PATHWAY)	NDC1, SMCR8, VAPB, WDR41, ATF6, EIF2AK3, ITPR3, NUP107, NUP133, NUP155, NUP98	6.7%	1.1E-3	ER Stress, defects in mRNA export, autophagy.
Envelope proteins and their potential roles in EDMD physiopathology (WIKIPATHWAYS)	LEMD3, ADCY9, EMD, LBR, SYNE3, SYN1, SYN2, TMPO	4.8%	6.2E-8	Interactions With F-actin, SUN, and nuclear lamins.
Cholesterol biosynthesis with skeletal dysplasia (WIKIPATHWAYS)	DHCR24, NSDHL, LBR	1.8%	1.3E-3	Errors in LBR and NSDHL are associated with Greenberg dysplasia and congenital hemidysplasia with ichthyosiform erythroderma.

Supplemental Table 2

Antibody Table D84G Paper

Antigen	Host	Dilution	Manufacturer	Cat #	Use
Stim1 N terminus	Rabbit poly	1:2000<br>1:250	Protein Tec	11565-1-AP	WB,<br>IF
Stim1 C terminus	Rabbit Poly	1:500	Sigma	S6197	WB
Sun2	Mouse mono	1:500<br>1:1000	Millipore	MABT880	WB<br>IF
Lamin A/C 4C11	Mouse mono	1:500	Cell Signalling	4777	WB<br>IF
PH2AX	Rabbit Mono	1:500	Cell Signaling	9718	IF
SERCA1	Mouse mono	1:1000	Thermo Fisher	VE121G9	IF
RyR	Mouse mono		Thermo Fisher	3925	WB
Calsequestrin	Mouse mono		Thermo Fisher	3913	WB
Sln	Rabbit Poly	1:1000	Millipore	ABT13	WB
cGAS	Rabbit Mono		Cell Signaling	31659	WB
STING1	Rabbit Mono		Cell Signaling	50494	WB
Histone 3	Rabbit Mono		Cell Signaling	9715s	WB
GAPDH	Rabbit Poly		Sigma	G9545	WB
					WB
Myosin type 1	Mouse monoclonal		Hybridoma	BAF8	IHC
Myosin type 2a	Mouse monoclonal		Hybridoma	SC71	IHC
Myosin Type 2b	Mouse monoclonal		Hybridoma	BFF3	IHC
SMAA	Mouse Mono	1:1000	Sigma	A2547	IF
